# Congenital central hypoventilation syndrome with hyperinsulinemia in an infant

**DOI:** 10.1186/1687-9856-2015-S1-P122

**Published:** 2015-04-28

**Authors:** Uma Ganti, Andrew Wilson, Maree Grant, Glynis Price, Shripada Rao, Martin de Bock

**Affiliations:** 1 Department of Endocrinology and Diabetes, Princess Margaret Hospital, Perth, WA, Australia; 2 Department of Paediatric Respiratory Medicine, Princess Margaret Hospital, Perth, WA, Australia; 3 Department of Neonatology, Princess Margaret Hospital, Perth, WA, Australia

## 

Congenital central hypoventilation syndrome (CCHS) is a rare disorder that typically presents in the newborn period and is characterized by alveolar hypoventilation and symptoms of autonomic nervous system dysregulation.

We describe an infant with CCHS who developed hyperinsulinism, which is an uncommon association. She was born by semi-elective Caesarean section at 37 weeks of gestation after a pregnancy complicated by polyhydramnios. Apgar scores were 7and 9 (at 1 and 5 min) and birth weight was 3550 g. She was intubated and ventilated within 24 hours for frequent apnoea’s and desaturations. Multiple extubation attempts on the following days failed because of progressive respiratory acidosis. She underwent tracheostomy at 2 months of age. CCHS was diagnosed in the first month of life, after a polyalanine repeat expansion of the PHOX2B gene (20/26genotype) was detected.

At 7 months of age, she was admitted to hospital with seizures associated with hypoglycaemia. A critical sample (glucose 2.5mmol/L) revealed an inappropriately raised plasma insulin of 3.0 mU/L, growth hormone 7mu/L, cortisol 470 mmol/L, and serum ketones <0.1g/L. Urine and serum metabolic screen was normal, as was an EEG. Diazoxide was commenced at 5 mg/kg/day along with hydrochlorothiazide at 1mg/kg/d. These relatively low doses were used in view of parental concerns of potential drug side effect of hirsutism. The dose had to be increased to 7.5mg/kg/day as hypoglycaemic episodes persisted. Capillary blood glucose testing and continuous glucose monitoring showed no further hypoglycaemia at this dose. Sequence analysis of the KCNJ11 and ABCC8 genes implicated in congenital hyperinsulinism was undertaken and no pathogenic mutation was found.

The mechanisms underlying abnormal glycaemia in CCHS are not fully understood. Multiple hypotheses have been postulated including the co-expression of the dopamine beta hydroxylase gene mutation with PHOX 2B mutation. Further, disordered autonomic homeostasis as seen in PHOX2B mutations is thought to impact the regulation of insulin and glucagon secretion.

Our case highlights the importance of considering hyperinsulinism in the differential diagnosis of hypoglycaemia in infants with CCHS.

Written informed consent was obtained from the patient's parent or guardian for publication of this abstract and any accompanying images. A copy of the written consent is available for review by the Editor of this journal.

